# Long-Term Maternal Mental Health after Spontaneous Preterm Birth

**DOI:** 10.1055/a-2182-4131

**Published:** 2023-11-01

**Authors:** Laura E. Janssen, Aranka R.C. Laarman, Elisabeth M. van Dijk-Lokkart, Tinka Bröring-Starre, Martijn A. Oudijk, Christianne J.M. de Groot, Marjon A. de Boer

**Affiliations:** 1 Department of Obstetrics, Reproduction and Development Research Institute, Amsterdam UMC, Vrije Universiteit Medical Center, Amsterdam, The Netherlands; 2 Department of Child and Adolescent Psychiatry and Psychosocial Care, Amsterdam UMC, Emma Childrens' Hospital, Vrije Universiteit Medical Center, Amsterdam, The Netherlands; 3 Department of Child and Adolescent Psychiatry and Psychosocial Care, Amsterdam UMC, Emma Childrens' Hospital, Amsterdam Medical Center, Amsterdam, The Netherlands; 4 Department of Obstetrics, Reproduction and Development Research Institute, Amsterdam UMC, Amsterdam Medical Centre, Amsterdam, The Netherlands

**Keywords:** spontaneous preterm birth, depression, depressive symptoms, anxiety, psychosocial distress

## Abstract

**Objective** The aim of this study is to investigate whether a history of spontaneous preterm birth (SPTB) is associated with maternal depressive and anxiety symptoms, or psychosocial distress in the fifth decade of life.

**Study Design** This is a secondary analysis of the PreCaris-study, a prospective observational study in which we included 350 women with a history of SPTB between 22 ^0/7^ and 36 ^6/7^ weeks of gestation and compared them to 115 women who had a term birth. Primary outcomes were the Depression and Anxiety scores measured using the Hospital Anxiety Depression Scale and Psychosocial distress assessed with the Distress Thermometer for Parents. Secondary outcomes were self-reported impact of the birth in daily life and psychosocial support after delivery.

**Results** After a median of 13 years after delivery, no significant differences were found in primary outcomes. Significantly more women with a history of SPTB reported that the birth still had impact in daily life; adjusted odds ratio: 2.46 (95% confidence interval: 1.35–4.48). A total of 57 (16.3%) women after SPTB reported to have needed professional psychosocial support after delivery but did not receive it. These women more often had a high Anxiety score ( *p* = 0.030), psychosocial distress ( *p* = 0.001), and influence of birth in daily life ( *p* = 0.000).

**Conclusion** There are no long-term effects on depressive and anxiety symptoms and psychosocial distress in women who experienced SPTB compared with women who had a full-term pregnancy. A significant part of the women who delivered preterm needed psychosocial support but did not receive it and were at higher risk of anxiety, psychosocial distress, and impact in daily life. We therefore recommend offering all women after SPTB psychosocial support after delivery.

**Key Points**

No long-term effects on depressive and anxiety symptoms and psychosocial distress after SPTB.

A total of 16.3% of the cases needed professional psychosocial support after delivery but did not receive it.

This subgroup was at higher risk of anxiety symptoms, psychosocial distress, and impact on daily life.

Preterm birth (PTB) remains a significant challenge in the field of obstetrics, as it continues to be the leading cause of neonatal morbidity and mortality globally. 1 According to the estimates from 2014, approximately 14.8 million infants, accounting for 10.6% of all live births worldwide, were born prematurely. 2 Spontaneous preterm birth (SPTB), characterized by delivery before 37 weeks of gestation due to either spontaneous contractions with intact membranes or spontaneous rupture of the membranes, accounts for approximately 65 to 75% of all PTBs. 3

PTB is associated with short-term complications, including respiratory distress syndrome, intraventricular hemorrhage, and necrotizing enterocolitis as well as long-term consequences, such as neurocognitive impairment. 4 5 Previous studies have shown that mothers of these children are at greater risk of psychological distress than mothers of term infants during infant hospitalization. 6 7 These elevated levels of maternal psychological distress may continue for months or even 2 years after hospital discharge. 8 9 10 11 12 Bener et al. showed that depression (29.4 vs. 17.3%) and anxiety (26.5 vs. 11.6%) were significantly more common among women with an SPTB compared with term birth within 6 months postpartum. 9 In addition, Bozzette et al. found that 16% of the mothers had elevated depressive symptoms when their children were 3 years of age. 13

Overall, having a preterm child appears to influence parental mental health, stress, and family functioning during early childhood. However, very little attention has been paid to long-term influence of PTB on the experience of psychological distress in women later in life. Treyvaud et al. showed that parents of preterm born children (gestational age [GA] < 30 weeks or birth weight < 1,250 g) reported higher levels of anxiety, depression symptoms, and levels of parenting stress 7 years after birth. 14 Bener et al. found a negative influence on the psychosocial and/or work environment of parents, 19 years after the PTB of their child. 9 However, this study is published in 2013 and based upon a cohort from 1983.

It is hypothesized that SPTB can cause stress, anxiety, and uncertainty in parents, both not only directly after pregnancy but also in the rest of their life. 15 The aim of our study was to investigate whether women with a history of SPTB had higher scores for depressive and anxiety symptoms 9 to 16 years after pregnancy.

## Materials and Methods

This is a secondary analysis of the PreCaris-study, a prospective observational study in which we included women with a history of SPTB, the cases, and compared them to women who had a term birth, the controls. SPTB was defined as PTB between 22 ^0/7^ and 36 ^6/7^ weeks starting with spontaneous contractions or spontaneous rupture of membranes. A history of term birth was defined as giving birth at or after 37 ^0/7^ weeks of gestation. The full list of exclusion criteria is provided in Supplementary Material S1 (available in the online version). Medical records were screened for inclusion and exclusion criteria, and eligible women were invited. All participants gave written informed consent prior to their inclusion in the study and received cardiovascular risk assessment at the Amsterdam UMC, location VU Medical Center and filled out questionnaires about physical and mental health.

Approval for the study was obtained from the Medical Ethics Committee of the VU University Medical Center in Amsterdam and from the hospital board of the Academic Medical Center Amsterdam (protocol approval: NL38972.029.12).

### Measures

Maternal mental health was assessed with the Hospital Anxiety and Depression Scale (HADS). The HADS is a 14-item self-report questionnaire, designed for screening of depressive and anxiety symptoms in nonpsychiatric patients. 16 The HADS is divided into two subscales: the depression (HADS-Depression) and anxiety subscale (HADS-Anxiety), both consisting of 7 items. The item scores range from 0 to 3; therefore, the scores of the anxiety and depression scales range from 0 to 21. A higher score indicates more depressive or anxiety symptoms. Maternal psychosocial distress was measured by the Distress Thermometer for Parents (DT-P), which is a well-validated, brief screening instrument that is used in clinical practice in the Netherlands to identify distress and everyday problems in parents of children with a chronic condition. 17 The DT-P ranges from 0 to 10, with a cutoff score of 4 for psychosocial distress, which consist of 29 individual items that reflect everyday problems in five domains (practical, social, emotional, physical, and cognitive). For each item, participants could indicate with “yes” or “no” if they experienced any of the problems in the last week, by which we could identify the sources of psychosocial distress.

We also asked whether the (spontaneous preterm) birth and events around it still have impact on daily life, which could be answered with “yes” or “no.” When answered with yes, participants could write down their explanation in an open text block. The answers were subdivided in four categories by 2 independent investigators: being too protective to your child, concerns about a disabled child, psychosocial problems, or a positive influence of the (preterm) birth. Next to that, we asked whether participants received psychosocial support after delivery, which could be answered with “yes” or “no and I did not need it” or “no but I needed it.”

### Outcomes

The primary outcomes were the median HADS-Depression and HADS-Anxiety scores, and psychosocial distress, defined as a score ≥4 on the DT-P. 17 We also investigated the incidence of elevated depressive symptoms, which was defined as a HADS-Depression score ≥ 11 and elevated anxiety symptoms defined as a HADS-Anxiety score ≥ 11, which indicates a suspected moderate-to-severe elevation. 16 Secondary outcomes were the incidence of impact of (spontaneous preterm) birth in daily life and psychosocial support after delivery.

### Statistical Analysis

Patient characteristics were examined using chi-square test, Fisher's exact test, independent samples *t* -test, or Mann–Whitney *U* -test. All calculations to obtain corresponding *p* -values were two-sided. The characteristics for continuous variables were presented as mean and standard deviation (SD), variables with a skewed distribution as median and interquartile range (IQR). Categorical variables were presented as percentages of numbers for corresponding group. A multivariable analysis was performed to adjust for potential confounders using hierarchical backward elimination, including covariates that were moderately associated with PTB ( *p* < 0.1). We performed a subgroup analysis based upon of the severity of SPTB: extreme (22 ^0/7^ –27 ^6/7^ weeks of gestation), very (28 ^0/7^ –31 ^6/7^ weeks of gestation), and moderate preterm (32 ^0/7^ –36 ^6/7^ weeks of gestation) and based upon birth weight: < 1,000, 1,000–1,500, and 1,500–2,500 g. In all analyses, a *p* -value < 0.05 was considered statistically significant. Data were analyzed using SPSS 22 software (Chicago, IL).

## Results

**Fig. 1:**
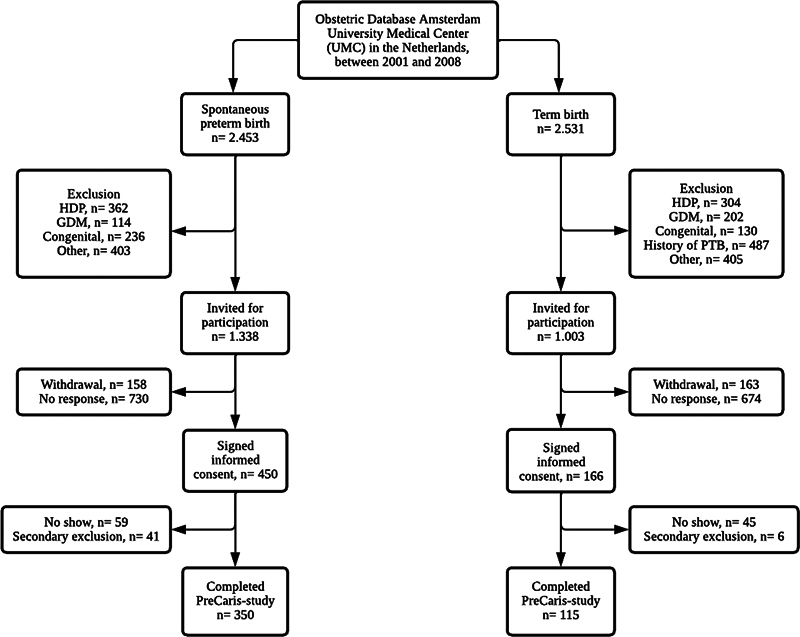
Flow chart of the study population. GDM, gestational diabetes mellitus; HDP, hypertensive disorder of pregnancy; PTB, preterm birth. Other: maternal death, renal disease, coagulation disorders, or Raynaud's syndrome.

In our original trial, a total of 350 cases and 115 controls underwent risk assessment, see the flowchart in Fig. 1 . Baseline characteristics at index pregnancy are shown in Table 1 . Cases were on average 1 year younger than controls ( *p* = 0.039). Most of the cases delivered moderate preterm. Years of follow-up and mean age was comparable between the groups. More cases were Caucasian ( *p* = 0.011) and had a low education compared with controls ( *p* = 0.010). There were no significant differences in obstetric history between the groups ( Table 2 ).

**Table 1:** Baseline characteristics at index pregnancy

Characteristics	SPTB ( *n* = 350)	Term birth ( *n* = 115)	*p* -Value
Age, y	31.2 ± 5.0	32.2 ± 4.7	0.039
Severity a			
Extremely preterm	82 (23.4)	–	–
Very preterm	108 (30.9)	–	–
Moderate preterm	160 (45.7)	–	–
Outcome			
Gestational age at delivery, wk	31.4 [28.3–35.3]	39.9 [38.9–40.9]	0.000
Cesarean	42 (12.0)	21 (18.3)	0.064
Birth weight, g	1,712 [1,188–2,539]	3,400 [3,145–3,703]	0.000
Fetal sex, boy	214 (61.1)	53 (46.1)	0.003
NICU administration	213 (60.9)	3 (2.6)	0.000
Perinatal death	39 (11.1)	0 (0)	0.000

Abbreviations: IQR, interquartile range; NICU, neonatal intensive care unit; SD, standard deviation; SPTB, spontaneous preterm birth.

Note: Values are mean ± SD or *n* (%) or are median [IQR].

a Extremely preterm: gestational age 22 ^0/7^ to 27 ^6/7^ weeks, very preterm: gestational age 28 ^0/7^ to 31 ^6/7^ weeks, moderate preterm: gestational age 32 ^0/7^ to 36 ^6/7^ weeks.

**Table 2:** Characteristics 9 to 16 years after pregnancy

Characteristics	SPTB ( *n* = 350)	Term birth ( *n* = 115)	*p* -Value
Age, y	44.6 ± 5.5	45.6 ± 5.6	0.128
Time post index pregnancy, y	13.0 ± 2.5	12.8 ± 2.6	0.560
Caucasian a	247 (70.6)	67 (58.3)	0.011
Current smoker	43 (12.3)	14 (12.2)	0.561
Education level b			0.020
Low	51 (14.6)	7 (6.1)	0.010
Intermediate	120 (34.3)	35 (30.4)	0.260
High	179 (51.1)	73 (63.5)	0.014
Obstetric history			
Miscarriage	128 (36.6)	33 (28.7)	0.076
Stillbirth	14 (4.0)	1 (0.9)	0.080

Abbreviations: SD, standard deviation; SPTB, spontaneous preterm birth.

Note: Values are mean ± SD or *n* (%).

a Country of birth of participant and the minimum of one parent or both parents despite participant in Europe, Western Asia, Central Asia, North Africa, and the Horn of Africa.

b Low: primary education, lower general secondary education; intermediate: high school, intermediate vocational education; high: preuniversity education, higher vocational education, and university.

The questionnaire results are shown in Table 3 . Median HADS-Depression and HADS-Anxiety score did not differ between the groups. Neonatal intensive care unit (NICU) admission, neonatal death, and previous PTB were not associated with HADS-scores. Psychological distress was present in approximately half of the cases and controls ( *p* = 0.365). Mean distress scores on practical, social, emotional, physical, and cognitive level did not differ between the groups. NICU admission and neonatal death were not associated with psychosocial distress.

**Table 3:** Primary and secondary outcomes spontaneous preterm birth versus term birth

Outcomes	SPTB ( *n* = 350)	Term birth ( *n* = 115)	Odds ratio (95% CI)	Adjusted odds ratio d (95% CI)
HADS-Depression score	3 [1–5]	3 [1–5]	–	–
HADS-Anxiety score	5 [3–8]	5 [3–7]	–	–
Elevated depression score a	17 (4.9)	6 (5.2)	0.93 (0.36–2.41)	1.19 (0.44–3.18)
Elevated anxiety score b	30 (8.6)	7 (6.1)	1.44 (0.62–3.39)	1.48 (0.62–3.52)
Psychosocial distress c	168 (48.0)	58 (50.4)	0.91 (0.60–1.38)	0.93 (0.60–1.44)
Self-reported impact daily life	93 (26.6)	15 (13.0)	2.41 (1.33–4.36) e	2.49 (1.37–4.54) e
Received psychosocial help	75 (21.4)	8 (7.0)	3.65 (1.70–7.82) e	3.74 (1.74–8.07) e
Needed but did not receive help	57 (16.3)	11 (9.6)	1.83 (0.93–3.64)	2.03 (1.01–4.06) e

Abbreviations: CI, confidence interval; IQR, interquartile range; HADS, Hospital Anxiety Depression Scale; SPTB, spontaneous preterm birth.

Note: Values are *n* (%) or median [IQR].

a Defined as HADS-Depression score ≥11.

b Defined as HADS-Anxiety score ≥11.

c Defined as psychosocial distress score ≥4 on the Distress Thermometer for Parents.

d Adjusted for country of birth and level of education.

e *p* -Value <0.05.

Significantly more cases reported that the birth still has impact in daily life. NICU admission and neonatal death significantly influenced the risk of experiencing impact in daily life (adjusted odds ratio [aOR]: 3.42; 95% confidence interval [CI]: 1.58–7.41). Of these women, most reported psychosocial problems ( *n* = 51, 59.3%), such as panic attacks, depression, sleeping problems, and recurrent thoughts of the (preterm) birth. Others mentioned problems in taking care of their disabled child ( *n* = 12, 12.9%) and being too protective to their child ( *n* = 10, 10.8%). However, not only negative impact was mentioned; 13 cases (14.0%) indicated that the PTB changed them into the person they are today, which had a positive outcome in their daily life, including less worrying about the little things and more feelings of strength, proudness, and thankfulness for life.

Among cases, a total of 75 (21.4%) women received professional psychosocial support after delivery, which was significantly more compared with controls ( *p* ≤ 0.001). Women with an extreme PTB received psychosocial help most often ( *n* = 33, 40.2%) compared with very ( *n* = 28, 25.9%) and moderate PTBs ( *n* = 14, 8.8%, *p* ≤ 0.001). Psychosocial support was more often given to participants experiencing neonatal loss ( *n* = 17, 43.6%) compared with participants with a living infant ( *n* = 66, 15.5%, *p* ≤ 0.001). Among cases, most ( *n* = 44, 58.7%) received psychosocial support from medical social workers during their stay in the hospital. Psychosocial support was less often given by a psychologist ( *n* = 25, 33.3%) or psychiatrist ( *n* = 6, 8.0%).

A total of 57 (16.3%) cases reported to have needed professional psychosocial support after delivery but did not receive it, consisting of 8 (9.8%) extreme PTB, 20 (18.5%) very PTB, and 29 (18.1%) moderate PTB ( *p* = 0.079). Neonatal death occurred in 3 (5.3%) cases and did not significantly alter the results. Women that needed psychosocial help but did not receive it, had elevated HADS-Anxiety scores ( *p* = 0.030), reported more psychosocial distress ( *p* = 0.001), and influence in daily life ( *p* = 0.000).

Subanalysis showed that a history of extreme PTB was associated with higher impact of birth in daily life compared with term birth. A history of very PTB was associated with elevated HADS-Anxiety scores and impact in daily life ( Table 4 ). Neonatal death did not significantly alter the results. Subanalysis based on birth weight is shown in Table 5 . After adjusting for neonatal death, birth weight between 1,000 and 1,500 g remained strongly associated with self-reported impact in daily life (aOR: 4.06, 95% CI: 2.20–7.48).

**Table 4:** Subanalysis based upon the severity of spontaneous preterm birth, compared with term birth

Outcomes	Extreme preterm ( *n* = 82)	Very preterm ( *n* = 108)	Moderate preterm ( *n* = 160)
Elevated depression score a	1.26 (0.32–4.95)	2.28 (0.60–8.63)	1.01 (0.34–3.07)
Elevated anxiety score b	1.22 (0.36–4.15)	3.14 (1.10–9.01) ^d^	1.17 (0.43–3.16)
Psychosocial distress c	0.94 (0.52–1.70)	0.76 (0.43–1.34)	1.05 (0.64–1.74)
Self-reported impact daily life	4.86 (2.35–10.07) d	3.14 (1.55–6.36) ^d^	1.20 (0.60–2.42)

Notes: Adjusted odds ratios for country of birth and level of education with 95% confidence interval. Extreme preterm = GA 22 ^0/7^ to 27 ^6/7^ weeks; very preterm = GA 28 ^0/7^ to 31 ^6/7^ weeks; moderate preterm = GA 32 ^0/7^ to 36 ^6/7^ weeks.

a Defined as HADS-Depression score ≥ 11.

b Defined as HADS-Anxiety score ≥ 11.

c Defined as psychosocial distress score ≥4 on the Distress Thermometer for Parents.

d *p* -Value <0.05.

**Table 5:** Subanalysis based upon birth weight compared with >2,500 g

Outcomes	<1,000 g ( *n* = 55)	1,000–1,500 g ( *n* = 94)	1,500–2,500 g ( *n* = 108)
Elevated depression score a	1.43 (0.35–5.77)	1.86 (0.58–6.04)	1.09 (0.36–3.38)
Elevated anxiety score b	0.42 (0.09–2.03)	2.00 (0.84–4.75)	1.17 (0.49–2.81)
Psychosocial distress c	0.70 (0.37–1.33)	0.75 (0.45–1.26)	0.77 (0.47–1.25)
Self-reported impact daily life	4.12 (2.03–8.35) d	4.01 (2.21–7.27) d	1.99 (1.08–3.68) d

Note: Adjusted odds ratios for country of birth and level of education with 95% confidence interval.

a Defined as HADS-Depression score ≥11.

b Defined as HADS-Anxiety score ≥11.

c Defined as psychosocial distress score ≥4 on the Distress Thermometer for Parents.

d *p* -Value < 0.05.

## Discussion

This observational cohort study investigated the long-term effects of SPTB on maternal mental health and psychosocial distress 9 to 16 years after pregnancy. After a median follow-up time of 13 years after SPTB we found that depressive, anxiety symptoms, and psychological distress were comparable to women after a term delivery. Yet, elevated anxiety scores were more common among women who delivered very preterm. A history of SPTB was significantly associated with the impact of the birth and events around it on daily life, which was reported most often among participants with a history of extreme PTB and birth weight between 1,000 and 1,500 g.

HADS-Depression and HADS-Anxiety scores were comparable with normative data in the Dutch population. 18 The incidence of moderate-to-severe elevated depression symptoms was low in both cases and controls and comparable with the study of Yaari et al. 19 Our results differ from the previous study performed by Treyvaud et al. which found higher levels of depression and anxiety, 7 years after delivery. 14 The differences could be explained by the longer follow-up period of our study, which was median 13 years compared with 7 years in the study of Treyvaud and colleagues. In contrast, we not only included women with extreme PTB and low birth weight, but all women with a history of SPTB. It is hypothesized that infants born extremely preterm or with low birth weight are often hospitalized in the NICU and are therefore at high risk for complications and mortality, suggesting that these mothers had a more traumatic and stressful postpartum period, which leads to higher HADS-scores. In our subanalysis, we found that a history of very PTB and not extreme PTB was significantly associated with elevated HADS-Anxiety. Birth weight was, after adjustment for neonatal death, not significantly associated with HADS-scores. This could be explained by the fact that mothers tend to experience resilience and posttraumatic growth after a traumatic event, which leads to lower HADS-scores among women with extreme PTB and birth weight below 1,000 g on the long term. 20 21 22

Psychosocial distress in both cases and controls was comparable with the incidence of distress in the Dutch female population. 18 In contrast to what we expected, subanalysis showed that the severity of SPTB and birth weight were not associated with psychosocial distress, although their infants are at higher risk for chronic conditions later in life. Our findings could be explained by the fact that late preterm infants primarily develop cognitive and behavioral problems. 22 23 These problems may be identified only later in life, during late childhood or adolescence, when social and academic demands increase. This could influence the maternal distress level on the long term, because studies suggesting that the quality of life of mothers is associated with the child's mental health and peer relationships and not particularly with the child's disabilities. 24 This explanation, together with the resilience and posttraumatic growth as mentioned before, is in line with our results suggesting that NICU admission and neonatal mortality were not predictive for elevated HADS-Depression, HADS-Anxiety, and psychosocial distress 13 years after SPTB.

In our study, a history of SPTB was significantly associated with impact of the birth and events around it in daily life. This is in line with previous studies, finding a significant family impact after PTB. 14 25 26 Our results showed that GA, neonatal birth weight, NICU admission, and neonatal death were strongly predictive for experiencing impact of the birth in daily life. This is in line with other studies, finding a higher impact in families of very low birth weight children who had high neonatal medical risk, compared with low neonatal medical risk. 27 28 29 Additionally, it is possible that these women still show symptoms of posttraumatic stress disorder (PTSD), given their traumatic experiences perinatal and early postpartum. 12 Our results showed that SPTB was associated with feelings of sadness and recurrent thoughts about the birth, which is suggestive for PTSD.

A total of 75 (21.4%) participants received psychosocial support after delivery, which was most often given by a medical social worker. In the participating hospitals of this study, it was not standard care to offer psychosocial support after delivery. Among cases, one out of six reported to have needed psychosocial support after delivery but did not receive it. The incidence of elevated HADS-scores was higher among these cases, as they reported more psychosocial distress and negative impact of their child's (pre)term birth in daily life.

## Strengths and Limitations

This is the first study that investigated maternal mental health and psychosocial distress on the long term in the overall group of women who experienced an SPTB, regardless of birth weight in comparison to a history of term birth. We performed a subanalysis to assess the influence of GA and birth weight on the HADS-Depression, HADS-Anxiety, and psychosocial distress. In addition, we performed an extensive assessment in a large cohort with a total of 465 participants.

Our study had some limitations that should be addressed. We did not adjust for the potential confounder prenatal or perinatal depression and neurodevelopmental disabilities of the child since it was outside the scope of the initial research. A history of depression and anxiety are known risk factors for developing PTB, and neurodevelopmental disabilities are a strong predictor of maternal mental health, distress, and impact in daily life. 30 31 Furthermore, impact in daily life was measured based on one single question, where participants could indicate “yes” or “no.” It was not measured with a validated questionnaire. Finally, the HADS and DT-P were filled out based on how participants felt the past week. Therefore, short-term mood variations could have led to a distorted view of the long-term effects on maternal mental health and psychosocial distress.

Risk assessment of our study was 9 to 16 years after their index pregnancy. Even though follow-up period did not differ between cases and controls, the guideline for active treatment of extremely preterm infants in the Netherlands was modified twice during the study period. In 2006, the guideline changed to active treatment of infants born by 25 ^0/7^ weeks and in 2010 lowered to 24 ^0/7^ weeks of gestation. Active treatment lowers neonatal death and could therefore influence HADS and DT-P scores, both in a negative and positive way.

## Conclusion

In conclusion, there are no long-term effects of SPTB on maternal depressive and anxiety symptoms and psychosocial distress 13 years after pregnancy. However, a history of SPTB was significantly associated with higher impact of birth in daily life, especially in mothers of children who were born extreme preterm and with a low birth weight. A significant part of the women who delivered preterm needed psychosocial support but did not receive it. This subgroup was at higher risk of developing anxiety symptoms, psychosocial distress, and reported more often impact of birth in daily life. We therefore recommend offering psychosocial support after delivery to all women after SPTB.

## References

[1] Blencowe H Cousens S Oestergaard M Z National, regional, and worldwide estimates of preterm birth rates in the year 2010 with time trends since 1990 for selected countries: a systematic analysis and implications Lancet 2012 379 9832 2162 2172 22682464 10.1016/S0140-6736(12)60820-4

[2] Lee A C Blencowe H Lawn J E Small babies, big numbers: global estimates of preterm birth Lancet Glob Health 2019 7 01 e2 e3 30389450 10.1016/S2214-109X(18)30484-4

[3] Goldenberg R L Culhane J F Iams J D Romero R Epidemiology and causes of preterm birth Lancet 2008 371 9606 75 84 18177778 10.1016/S0140-6736(08)60074-4 PMC7134569

[4] Bhutta A T Cleves M A Casey P H Cradock M M Anand K JS Cognitive and behavioral outcomes of school-aged children who were born preterm: a meta-analysis JAMA 2002 288 06 728 737 12169077 10.1001/jama.288.6.728

[5] Platt M J Outcomes in preterm infants Public Health 2014 128 05 399 403 24794180 10.1016/j.puhe.2014.03.010

[6] Meyer E C Garcia Coll C T Seifer R Ramos A Kilis E Oh W Psychological distress in mothers of preterm infants J Dev Behav Pediatr 1995 16 06 412 417 8746550

[7] Miles M S Burchinal P Holditch-Davis D Brunssen S Wilson S M Perceptions of stress, worry, and support in Black and White mothers of hospitalized, medically fragile infants J Pediatr Nurs 2002 17 02 82 88 12029601 10.1053/jpdn.2002.124125

[8] Garel M Dardennes M Blondel B Mothers' psychological distress 1 year after very preterm childbirth. Results of the EPIPAGE qualitative study Child Care Health Dev 2007 33 02 137 143 17291317 10.1111/j.1365-2214.2006.00663.x

[9] Bener A Psychological distress among postpartum mothers of preterm infants and associated factors: a neglected public health problem Br J Psychiatry 2013 35 03 231 236 10.1590/1516-4446-2012-0821 24142082

[10] Holditch-Davis D Santos H Levy J Patterns of psychological distress in mothers of preterm infants Infant Behav Dev 2015 41 154 163 26495909 10.1016/j.infbeh.2015.10.004 PMC4654120

[11] Gray P H Edwards D M Gibbons K Parenting stress trajectories in mothers of very preterm infants to 2 years Arch Dis Child Fetal Neonatal Ed 2018 103 01 F43 F48 28659361 10.1136/archdischild-2016-312141

[12] Ouwendijk-Andréa M Bröring-Starre T Molderink A C Laarman C ARC Oostrom K J van Dijk-Lokkart E M Parental emotional distress after discharge from the neonatal intensive care unit: a pilot study Early Hum Dev 2020 140 104892 31715521 10.1016/j.earlhumdev.2019.104892

[13] Bozzette M Holditch-Davis D A preliminary study of depressive symptoms in mothers of 3-year-old prematurely born children Child Health Care 2015 44 01 54 68 25750472 10.1080/02739615.2013.876539 PMC4349490

[14] Treyvaud K Lee K J Doyle L W Anderson P J Very preterm birth influences parental mental health and family outcomes seven years after birth J Pediatr 2014 164 03 515 521 24359937 10.1016/j.jpeds.2013.11.001 PMC3950307

[15] Kusters C DJ van der Pal S M van Steenbrugge G J den Ouden L S Kollée L AA The impact of a premature birth on the family; consequences are experienced even after 19 years [in Dutch] Ned Tijdschr Geneeskd 2013 157 25 A5449 23777961

[16] Snaith R P The Hospital Anxiety And Depression Scale Health Qual Life Outcomes 2003 1 29 12914662 10.1186/1477-7525-1-29 PMC183845

[17] Haverman L van Oers H A Limperg P F Development and validation of the distress thermometer for parents of a chronically ill child J Pediatr 2013 163 04 1140 600 23910979 10.1016/j.jpeds.2013.06.011

[18] Spinhoven P Ormel J Sloekers P P Kempen G I Speckens A E Van Hemert A M A validation study of the Hospital Anxiety and Depression Scale (HADS) in different groups of Dutch subjects Psychol Med 1997 27 02 363 370 9089829 10.1017/s0033291796004382

[19] Yaari M Treyvaud K Lee K J Doyle L W Anderson P J Preterm birth and maternal mental health: longitudinal trajectories and predictors J Pediatr Psychol 2019 44 06 736 747 30977828 10.1093/jpepsy/jsz019 PMC7967874

[20] Bonanno G A Loss, trauma, and human resilience: have we underestimated the human capacity to thrive after extremely aversive events? Am Psychol 2004 59 01 20 28 14736317 10.1037/0003-066X.59.1.20

[21] Singer L T Fulton S Kirchner H L Longitudinal predictors of maternal stress and coping after very low-birth-weight birth Arch Pediatr Adolesc Med 2010 164 06 518 524 20530301 10.1001/archpediatrics.2010.81 PMC10222517

[22] Talge N M Holzman C Wang J Lucia V Gardiner J Breslau N Late-preterm birth and its association with cognitive and socioemotional outcomes at 6 years of age Pediatrics 2010 126 06 1124 1131 21098151 10.1542/peds.2010-1536

[23] Shah P E Robbins N Coelho R B Poehlmann J The paradox of prematurity: the behavioral vulnerability of late preterm infants and the cognitive susceptibility of very preterm infants at 36 months post-term Infant Behav Dev 2013 36 01 50 62 23261789 10.1016/j.infbeh.2012.11.003 PMC4235990

[24] Wolke D Baumann N Busch B Bartmann P Very preterm birth and parents' quality of life 27 years later Pediatrics 2017 140 03 e20171263 28798147 10.1542/peds.2017-1263

[25] Saigal S Burrows E Stoskopf B L Rosenbaum P L Streiner D Impact of extreme prematurity on families of adolescent children J Pediatr 2000 137 05 701 706 11060538 10.1067/mpd.2000.109001

[26] Saigal S Pinelli J Streiner D L Boyle M Stoskopf B Impact of extreme prematurity on family functioning and maternal health 20 years later Pediatrics 2010 126 01 e81 e88 20530081 10.1542/peds.2009-2527

[27] Singer L T Fulton S Kirchner H L Parenting very low birth weight children at school age: maternal stress and coping J Pediatr 2007 151 05 463 469 17961686 10.1016/j.jpeds.2007.04.012 PMC10228568

[28] Taylor H G Klein N Minich N M Hack M Long-term family outcomes for children with very low birth weights Arch Pediatr Adolesc Med 2001 155 02 155 161 11177090 10.1001/archpedi.155.2.155

[29] Drotar D Hack M Taylor G Schluchter M Andreias L Klein N The impact of extremely low birth weight on the families of school-aged children Pediatrics 2006 117 06 2006 2013 16740842 10.1542/peds.2005-2118

[30] Monroe S M Slavich G M Gotlib I H Life stress and family history for depression: the moderating role of past depressive episodes J Psychiatr Res 2014 49 90 95 24308926 10.1016/j.jpsychires.2013.11.005 PMC3918432

[31] Singer G HS Meta-analysis of comparative studies of depression in mothers of children with and without developmental disabilities Am J Ment Retard 2006 111 03 155 169 16597183 10.1352/0895-8017(2006)111[155:MOCSOD]2.0.CO;2

