# Supplementary material for: Long-Term Maternal Mental Health after Spontaneous Preterm Birth
Source: Am J Perinatol. 2023 Nov 1;41(Suppl 1):e2893–900. doi: 10.1055/a-2182-4131 (PMC11150068; doi:10.1055/a-2182-4131)
Supplement: Supplementary file 1 — Supplementary Material [file 10-1055-a-2182-4131-s22may1268.pdf]

Supplementary Material S1 Full list of exclusion criteria of the study population

For both cases and controls
• Age <18 years at the time of pregnancy
• Multiple pregnancy
• Chronic hypertension before pregnancy or hypertension in the first 20 weeks of pregnancy
• Hypertensive disorders in any pregnancy
• Uterine anomaly
• History of conization of the cervix
• Cardiovascular disease before pregnancy
• Diabetes mellitus before pregnancy or gestational diabetes during the index pregnancy
• Renal disease
• Coagulation disorders
• History of pregnancy complicated by fetal anomalies
• Raynaud's syndrome
• Currently pregnant
• Pregnancy in the last 6 months
• Currently breastfeeding
For cases
• Iatrogenic preterm birth in any pregnancy
For controls
• A history of preterm birth, either spontaneous or iatrogenic in any pregnancy

 © 2023. The Author(s).
